# Transmission Characteristics of Variably Protease-Sensitive Prionopathy

**DOI:** 10.3201/eid2012.140548

**Published:** 2014-12

**Authors:** Silvio Notari, Xiangzhu Xiao, Juan Carlos Espinosa, Yvonne Cohen, Liuting Qing, Patricia Aguilar-Calvo, Diane Kofskey, Ignazio Cali, Laura Cracco, Qingzhong Kong, Juan Maria Torres, Wenquan Zou, Pierluigi Gambetti

**Affiliations:** Case Western Reserve University, Cleveland, Ohio, USA (S. Notari, X. Xiao, Y. Cohen, L. Qing, D. Kofskey, I. Cali, L. Cracco, Q. Kong, W. Zou, P. Gambetti);; Instituto Nacional de Investigación y Tecnología Agraria y Alimentaria, Madrid, Spain (J.C. Espinosa, P. Aguilar-Calvo, J.M. Torres)

**Keywords:** transgenic mice, transmissibility, humans, VPSPr, prions and related diseases

## Abstract

Variably protease-sensitive prionopathy (VPSPr), a recently identified and seemingly sporadic human prion disease, is distinct from Creutzfeldt-Jakob disease (CJD) but shares features of Gerstmann-Sträussler-Scheinker disease (GSS). However, contrary to exclusively inherited GSS, no prion protein (PrP) gene variations have been detected in VPSPr, suggesting that VPSPr might be the long-sought sporadic form of GSS. The VPSPr atypical features raised the issue of transmissibility, a prototypical property of prion diseases. We inoculated VPSPr brain homogenate into transgenic mice expressing various levels of human PrP (PrP^C^). On first passage, 54% of challenged mice showed histopathologic lesions, and 34% harbored abnormal PrP similar to that of VPSPr. Surprisingly, no prion disease was detected on second passage. We concluded that VPSPr is transmissible; thus, it is an authentic prion disease. However, we speculate that normal human PrP^C^ is not an efficient conversion substrate (or mouse brain not a favorable environment) and therefore cannot sustain replication beyond the first passage.

## Abstract 2

This disease is transmissible and thus an authentic prion disease.

Prion diseases include a variety of animal and human conditions that might be sporadic, inherited, or acquired by infection. Despite this diversity, most prion diseases are thought to share the same pathogenetic mechanism whereby the cellular prion protein (PrP^C^) is templated into an abnormal and pathogenic conformer, commonly identified as scrapie PrP (PrP^Sc^) (*1*). Therefore, conversion by templating is the basic mechanism that causes disease and sustains disease transmission among humans (*2*). However, data have shown that propagation, disease manifestation, and transmissibility might occur separately in various ways (*3*,*4*).

The phenotype of human prion diseases is highly heterogeneous. This characteristic is largely due to the variable genotype at codon 129, the site of a methionine/valine (MV) polymorphism in the human PrP gene, and the molecular characteristics of the associated PrP^Sc^ (*5*). A further major distinction that has been applied to human prion diseases for years is based on the experimental transmissibility of these diseases to hosts thought to be permissive for exogenous human PrP^Sc^ (*6*). Based on this principle, the sporadic form of Creutzfeldt-Jakob disease (sCJD) belongs to the transmissible disease group, whereas most of the Gerstmann-Sträussler-Scheinker diseases (GSS), a group comprising exclusively inherited forms, were considered difficult to transmit or not transmissible (*7*–*10*). However, an increasing number of findings have challenged this distinction. Replication of infectious PrP^Sc^ occurs in the absence of clinical signs in the host, or even in the absence of detectable disease, by histologic and Western blot (WB) examinations. Yet, infectivity can be demonstrated in subsequent passages to more susceptible hosts (*9*,*11*,*12*). Finally, disease transmissibility has recently been demonstrated for a subtype of GSS previously thought to be nontransmissible (*8*,*10*,*13*).

In 2008 and 2010, we introduced a novel human prion disease, presumably sporadic, that we named variably protease-sensitive prionopathy (VPSPr) (*14*–*16*). VPSPr differs from sCJD in several aspects. The clinical presentation and the frequent slow progression evoke the features of atypical dementias, such as frontotemporal dementia, diffuse Lewy body disease, or normal pressure hydrocephalus (*17*). The flocculent PrP immunostaining pattern, which includes the frequent presence of PrP peculiar amyloid plaques, differs from those of other sporadic prion diseases. However, the most distinctive VPSPr feature rests on the characteristics of the associated PrP^Sc^, especially the electrophoretic profile and resistance to the commonly used protease, such as proteinase K (PK) (*14*,*15*,*17*). VPSPr-associated, PK-resistant PrP^Sc^ (resPrP^Sc^) forms a distinctive 5-band electrophoretic profile, comprising various fragments truncated at the N terminal and at least 1 fragment truncated at both N and C terminals, which consequently lacks the GPI (glycosylphosphatidylinositol) anchor (*14*). Furthermore, the N-truncated fragments do not include the diglycosylated PrP^Sc^ isoforms but only 1 of 2 monoglycosylated and the unglycosylated isoforms (*18*), and upon cleavage of the glycans, these fragments form 3 PK-resistant WB bands in VPSPr preparations. In contrast, the electrophoretic profile of sCJD resPrP^Sc^ is characterized by 3 bands, including diglycosylated, 2 monoglycosylated, and unglycosylated isoform, all of which harbor the GPI anchor and form only 1 band after glycan removal (*19*). However, VPSPr also occasionally displays small amounts of typical 3 electrophoretic band–forming resPrP^Sc^ on WB of basal ganglia and other deep cerebral structures (*14*). On the whole, the VPSPr 5-band ladder profile is more akin to the electrophoretic profile of PrP^Sc^ in most GSS subtypes, although contrary to GSS, no mutation in the PrP gene open reading frame has been observed in VPSPr (*14*–*16*). Given this similarity, Zou et al. hypothesized that VPSPr is the sporadic form of GSS (*15*). Like sCJD, VPSPr affects persons harboring each of 3 PrP^C^ 129 genotypes: methionine and valine homozygosity (MM, VV) and MV heterozygosity. The 3 genotypic subtypes of VPSPr differ slightly in clinical presentation, duration, histopathologic features, and PK resistance of the PrP^Sc^ (*15*).

The similarity in the PrP^Sc^ electrophoretic profiles with GSS, which was regarded as not transmissible, called into question the transmissibility of VPSPr and prompted use of the term prionopathy rather than prion disease. The present study addresses this issue. Transgenic (Tg) mice expressing various levels of human PrP^C^ harboring methionine (M) or valine (V) at position 129 underwent intracranial inoculation with brain homogenates (BH) from several human case-patients with VPSPr associated with each of the three 129 genotypes (*15*). Surprisingly, VPSPr was transmitted as an asymptomatic disease characterized by focal accumulation of VPSPr-like PrP^Sc^ in the first passage, but no prion disease could be demonstrated in the second passage.

## Materials and Methods

### VPSPr Case-Patients and Controls

We conducted this study during 2007–2013. Twelve case-patients with VPSPr (6 with VPSPr-129VV, 4 with VPSPr-129MV, and 2 with VPSPr-129MM) provided the inocula used in the first passage. The second passage inocula were obtained from Tg mice challenged at first passage with BH from frontal cortex and putamen of 1 case-patient with VPSPr-129VV and 1 with VPSPr-129MM. Two sets of experiments were conducted to generate negative controls by using Tg mice from the same lines: 1) age-matched Tg mice that were not inoculated and 2) age-matched Tg mice challenged with noninfectious BH from mice of the same genetic background. Positive controls were obtained from the same 129M and 129V Tg mice lines inoculated with BH from 5 subtypes of sCJD, sCJDMM1, sCJDMM2, sCJDVV1, sCJDVV2, and sCJDMV2, maintaining the host/donor homology of codon 129. Finally, six 129V Tg mice were euthanized 35 days postinoculation (dpi) with VPSPr-129VV, and the presence of the inoculum was searched by immunohistochemistry and immunoblotting.

### Tg Mice

Four mouse lines with different PrP^C^ expression levels were used. The first line was a Tg362 mouse line expressing human PrP^C^-129V on a mouse PrP^C^ null background at ≈8-fold normal human brain levels, hereafter identified as Tg(HuPrP129V)×8. This line was generated following previously described methods (*20*). For the next 2 lines,Tg40 and Tg21 were generated by replacing the mouse PrP open reading frame in the murine half-genomic PrP clone in plasmid pHGPRP (*21*) with that of human PrP-129M or human PrP-129V as reported (*22*). The generated Tg mice harbored a wild-type FVB background and bred with FVB/PrP null mice (*23*) to obtain Tg mice in mouse PrP null FVB background. For the fourth line, the generated Tg40 mouse line expressing human PrP^C^-129M at ≈1-fold normal mouse brain level, identified as Tg(HuPrP129M)×1, this was then self-bred to obtain the Tg40h mouse line. The generated Tg40h expressing human PrP^C^-129M at ≈2-fold normal mouse brain levels, was identified as Tg(HuPrP129M)×2. The Tg21 mouse line expressing human PrP^C^-129V at ≈3-fold normal mouse brain levels was identified as Tg(HuPrP129V) × 3.

### Intracerebral Inoculations

Ten percent or 1% BH in 5% glucose or phosphate-buffered saline were generated from the frontal cortex, occipital cortex, or putamen. It was inoculated intracerebrally according to procedures previously described (*20*,*22*).

### Ethical Considerations

We conducted animal experiments in strict accordance with the recommendations in the guidelines of the Code for Methods and Welfare Considerations in Behavioral Research with Animals (directive 86/609EC). All efforts were made to minimize suffering. Experiments were approved by the Committee on the Ethics of Animal Experiments of the Spanish National Instutute for the Agricultural and Food Research and Technology; permit no. CEEA 2011/046.

### Histology and Immunohistochemistry

Standard histologic and immunohistochemical examinations were conducted at 4 brain levels. These were basal ganglia (bregma + ≈0.14 mm), thalamus (bregma – ≈1.46 mm), posterior hippocampus (bregma – ≈2.92 mm), and anterior cerebellum (bregma – ≈6.00 mm), on paraffin sections stained with hematoxylin and eosin or probed with the 3F4 antibody to the prion protein (PrP) (1:1,000–1,400) (*24*).

### Immunochemistry

Conventional immunoblotting was conducted on frozen brain tissues homogenized in glucose solution to make 10% BH. To detect PK-resistant PrP^Sc^, we mixed the 10% BH with an equal volume of 2× lysis buffer. BH was treated with different amounts of PK (Sigma Chemical Co., St. Louis, MO, USA) ranging from 0 μg/mL to 50 μg/mL. The samples untreated or treated with PK, equivalent to 0.5–2 mg of wet tissues, were loaded onto 15% Tris-HCl Criterion precast gels (Bio-Rad Laboratories, Hercules, CA, USA) for SDS-PAGE (sodium dodecyl sulfate polyacrylamide gel electrophoresis) and immunoblotted with the widely used PrP antibody 3F4 against human PrP106–112 (*15*) or 1E4 against human PrP97–105 (*25*) (Cell Sciences, Inc., Canton, MA, USA). When required, PrP was deglycosylated with PNGase F (New England Biolabs, Beverly, MA, USA) before immunoblotting following the manufacturer’s instructions.

## Results

### First Passage

#### Histologic and Immunohistochemical Analyses

We found that 54% of the Tg mice belonging to 3 lines expressing 129V or 129M human PrP showed histologic abnormalities after inoculation of BH from 6 persons with symptomatic VPSPr (inoculum from 1 presymptomatic but histopathologically and immunochemically confirmed person with VPSPr did not transmit) (Table 1). All 30 positive Tg mice were asymptomatic until they were culled, after incubation periods of up to 800 days. Although the low number of mice inoculated with VPSPr-129MM made the comparison difficult, no major differences in the rate of transmission were detected between Tg(HuPrP129V) mice challenged with VPSPr-129VV and Tg(HuPrP129M) mice challenged with VPSPr-129MM. Similarly, we detected no significant difference in plaque prevalence between Tg mice expressing PrP^C^ at levels ×8 and ×3 normal (Table 1). Affected Tg mice were observed only when the 129 genotype of the VPSPr inoculum matched that of the host (Table 2); that is, neither positive Tg(HuPrP129M) nor Tg(HuPrP129V) mice were observed after inoculation with VPSPr-129MV and VPSPr-129VV or VPSPr-129MV BH, respectively. However, VPSPr-129MM transmission to Tg(HuPrP129V) was not tested, and Tg(HuPrP129M)×1, but not Tg(HuPrP129M)×2, was used for transmission of VPSPr-129VV.

**Table 1:** First passage VPSPr inoculations to Tg(HuPrP) harboring the same PrP 129 genotype as the inoculum*

Inoculum	Tg(HuPrP)	No. with clinical signs/total	Histology, immunohistochemistry			PrP^Sc^ Western blot	
			No. positive/total	Dpi positive/total†		No. positive/total	Dpi positive/total
VPSPr-129VV 1st‡	(129V)×8§	0/11	3/9	741 ± 76/741 ± 55		4/11	768 ± 74/733 ± 71
VPSPr-129VV 2nd		0/17	10/12	711 ± 65/722 ± 59		6/17	635 ± 204/682 ± 131
VPSPr-129VV 3rd		0/11	3/8	669 ± 92/651 ± 89		3/10	642 ± 157/604 ± 140
VPSPr-129VV 4th	(129V)×3	0/9	7/9	655 ± 67/642 ± 104		NA	NA
VPSPr-129VV 5th		0/6	3/6	541 ± 96/642 ± 76		NA	NA
VPSPr-129MM 1st	(129M)×2	0/6	4/6	678 ± 90/590 ± 154		NA	NA
VPSPr-129MM 2nd (presymptomatic)		0/6	0/6	0/746 ± 59		NA	NA

Dpi, days postinoculation; M, methionine; V, valine; NA, not available; VPSPr, variably protease-sensitive prionopathy associated with homozygous V or M at codon 129 of the PrP gene (VPSPr-129VV or VPSPr-129MM). †Dpi of positive mice compared with dpi of all mice (including positive mice). Each value represents the mean ± SD. ‡Identifies the VPSPr case-patients providing the inoculum, i.e., inocula were obtained from 5 case-patients with VPSPr-129VV and 2 with VPSPr-129MM. §Refers to the presence of V or M at PrP residue 129 and expression level indicated as × normal.

**Table 2:** First passage VPSPr inoculations to Tg(HuPrP) mice harboring different PrP 129 genotypes from those of the inocula*

Inoculum	Tg(HuPrP)	No. with clinical signs/total	Histology, immunohistochemistry			PrP^Sc^ Western blot	
			No. positive/total	Dpi positive/total†		No. positive/total	Dpi positive/total
VPSPr-129VV 6th‡	(129M)×1§	0/5	0/5	0/643 ± 71		NA	NA
VPSPr-129VV 4th		0/4	0/4	0/659 ± 55		NA	NA
VPSPr-129VV 5th		0/5	0/5	0/757 ± 57		NA	NA
VPSPr-129MV 1st	(129M)×2	0/9	0/9	0/745 ± 70		NA	NA
VPSPr-129MV 2nd		0/6	0/6	0/582 ± 101		NA	NA
VPSPr-129MV 3rd		0/4	0/4	0/694 ± 153		NA	NA
VPSPr-129MV 4st	(129V)×8	0/6	0/6	0/586 ± 68		NA	NA
VPSPr-129MV 3rd		0/7	0/7	0/681 ± 102		NA	NA

Dpi, days postinoculation; M, methionine; V, valine; NA, not available; VPSPr, variably protease-sensitive prionopathy associated with homozygosity V or M at codon 129 of the PrP gene (VPSPr-129VV or VPSPr-129MM). †Dpi of positive mice compared with dpi of all mice (including positive mice). Each value represents the mean ± SD. ‡Identifies the VPSPr case-patients providing the inoculum, i.e., inocula were obtained from 5 case-patients with VPSPr-129VV and 2 with VPSPr-129MM. §Refers to the presence of V or M at PrP residue 129 and expression level indicated as × normal.

**Figure 1:**
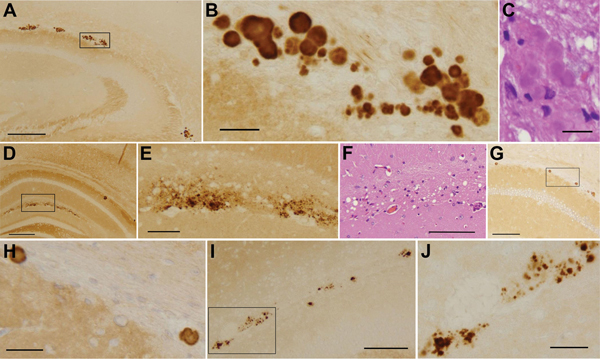
Histologic and immunohistochemical findings from a study of the transmission characteristics of VPSPr. A‒E). Two types of lesions typically observed in Tg(HuPrP129V)×8 mice. A‒C) Plaques are most often located at the border between the alveus of the hippocampus and the corpus callosum, often forming aggregates distributed in a row. They can be tightly aggregated or partially fused generating multicore plaques. PrP immunostaining (A, B) shows well-formed plaques surrounded by PrP deposits that appear in various stages of aggregation at HE. C), where plaque cores appear smooth, lacking the spiny appearance of typical kuru plaques. D‒F) The lesion of the second type consists of PrP granular deposits (D and E) co-localized with spongiform degeneration in the layer lacunosum moleculare of the hippocampus (F). G‒J) Plaques in Tg(HuPrP129V)×3 mice were fewer but similar in location and appearance to those of Tg(HuPrP129V)×8. In contrast, PrP aggregates generally appeared to be loose and formed fewer real plaques in Tg(HuPrP129M)×2–positive mice (I and J). PrP monoclonal antibody 3F4. The boxes in panels A, D, G, and I mark the exact areas that are shown at higher magnification in panels B, E, H, and J, respectively. PrP, prion protein; VPSPr, variably protease-sensitive prionopathy; Tg, transgenic. Scale bars in A and E = 250 μm. Scales bar in B, C, H, and J = 25 μm. Scale bar in D = 500 μm. Scale bars in F, G, and I = 100 μm.

**Figure 2:**
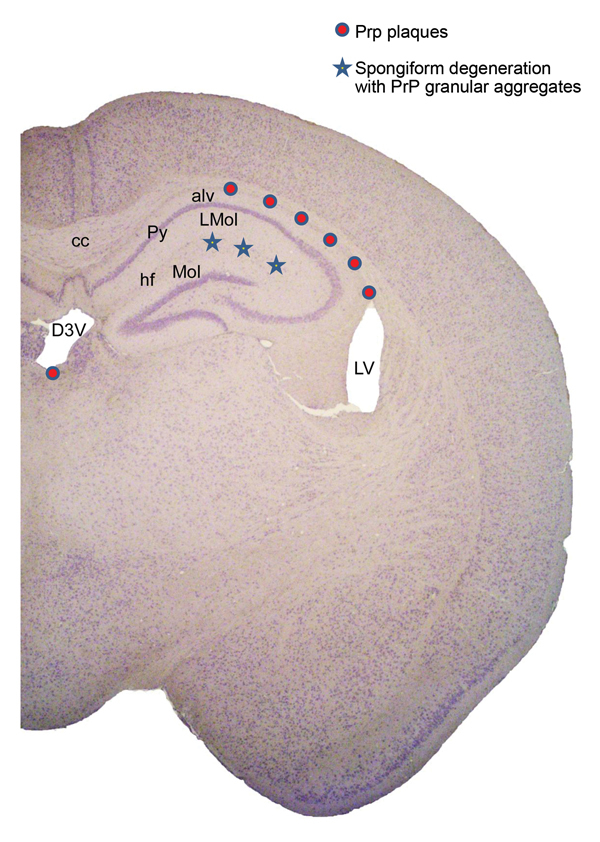
Representation of lesion topography in brain of positive Tg(HuPrP129V)×8 mice inoculated with VPSPr-129VV brain homogenate. alv, alveus of hippocampus; cc, corpus callosum; D3V, dorsal third ventricle; hf, hippocampal fissure; L mol, lacunosum molecular layer; LV, lateral ventricle; Mol, molecular layer dentate gyrus; Py, pyramidal cell layer of hippocampus.

The histologic lesions included 2 types. The most common lesion consisted of individual or aggregates of prion plaques often in a row preferentially located immediately below the corpus callosum at the border with the alveus of the hippocampus, which might represent the subependymal region of the lateral ventricles. We occasionally saw individual plaques in other periventricular regions but rarely in the parenchyma (Figures 1, 2). The lesion of the second type consisted of focal spongiform degeneration involving the layer lacunosum moleculare of the hippocampus (Figure 1). This lesion was seen almost exclusively in Tg(HuPrP129V)×8 and predominantly after inoculation of VPSPr BH from the basal ganglia. PrP immunostaining intensely enhanced the plaques and demonstrated PrP granular deposits in the focal areas of spongiform degeneration. Although plaques appeared very compact in Tg(HuPrP129V) mice inoculated with VPSPr-129VV, plaques in Tg(HuPrP129M) mice after VPSPr-129MM inoculation were less well formed and were often replaced by loose aggregates of irregular granules (Figure 1). The topographic distribution of the plaque deposits and focal spongiform change are shown in Figure 2.

Neither prion-related lesions nor PrP^Sc^ were observed in 23 age-matched Tg(HuPrP129)V×8 mice that had not been inoculated or had undergone inoculations with noninfectious BH (Table 3). As expected, the 3 Tg(HuPrP) mouse lines used for VPSPr transmission easily transmitted sCJD subtypes that were 129 genotypically compatible with the host PrP, indicating that the Tg mice used for VPSPr transmission are competent to propagate classical human prion diseases (Table 3). No PrP immunostaining was detected in the 6 Tg(HuPrP129V)×8 35 dpi with VPSPr-129VV BH.

**Table 3:** Control studies of VPSPr inoculations to Tg(HuPrP)*

Inoculum	Tg(HuPrP)	No. with clinical signs/total	Histology, immunohistochemistry			PrP^Sc^ Western blot	
			No. positive/total	Dpi positive/total†		No. positive/total	Dpi positive/total
Negative controls							
None	(129V)×8‡	0/15	0/9	0/721 ± 43		0/4	0/704 ± 43
Noninfectious		0/17	0/8	0/744 ± 15		0/5	0/749 ± 112
Positive controls							
sCJDVV2§	(129V)×8	6/6	3/3	223 ± 13/223 ± 13		6/6	223 ± 11/223 ± 11
sCJDVV1		7/7	7/7	353 ± 12/353 ± 12		7/7	353 ± 12/353 ± 12
sCJDMM1	(129M)×2	3/3	3/3	183 ± 22/183 ± 22		3/3	183 ± 22/183 ± 22
sCJDMM2		3/3	3/3	609 ± 139/609 ± 139		3/3	609 ± 139/609 ± 139
sCJDMV2	(129V)×3	6/6	6/6	312 ± 4/312 ± 4		NA	NA

Dpi, days postinoculation; M, methionine; V, valine; NA, not available; VPSPr, variably protease-sensitive prionopathy; sCJD sporadic Creutzfeldt-Jakob disease. †Dpi of positive mice compared with dpi of all mice (including positive mice). Each value represents the mean ± SD. ‡Refers to the presence of V or M at PrP residue 129 and expression level indicated as × normal. §Identifies the sCJD case-patients providing the inoculum.

#### WB Analysis

**Figure 3:**
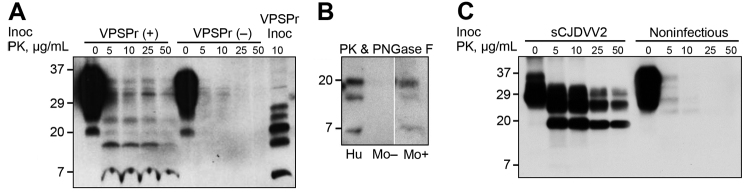
Western blot characteristics of PrP^Sc^ recovered from brain of VPSPr-inoculated Tg mice and controls. A) BH treated with increasing amounts of PK show PK-resistant PrP^Sc^ fragments with a ladder-like electrophoretic profile in positive VPSPr-inoculated mice, VPSPr (+), even at high concentrations of PK (50 μL/mL). In contrast, nonspecific bands are seen in negative VPSPr-inoculated mice, VPSPr (‒). The banding pattern in VPSPr (+) roughly recapitulates that of the PK-treated PrP^Sc^ from the VPSPr inoculum (VPSPr Inoc). B) Positive Tg mice BH treated with PK (25 μg/mL) and PNGase F show 3 PrP^Sc^ bands migrating at ≈20 kDa, ≈17 kDa, and ≈7 kDa (Mo +), replicating those of similarly treated VPSPr inoculum (Hu). No bands can be detected in the negative Tg mice (Mo ‒). (All 3 preparations were run on the same gel, but unnecessary lanes were removed). C) Tg mice inoculated with sCJDVV2 BH (from sCJD homozygous valine harboring PrP^Sc^ type 2) or inoculated with noninfectious BH used as positive and negative controls exhibit typical PK-resistant PrP^Sc^. Tg(HuPrP129V)×8 BH and monoclonal antibody 1E4 were used in all Western blot tests. Approximate molecular masses are in kDa. BH, brain homogenate; PK, proteinase K; PrP^Sc^, scrapie prion protein; sCJD, sporadic Creutzfeldt-Jakob disease; VPSPr, variably protease-sensitive prionopathy; Tg, transgenic.

Thirteen (34%) of the 38 VPSPr-inoculated Tg(HuPrP129V)×8 mice examined showed a definitively positive WB, despite the mouse BH treatment with a wide range of PK to maximize the recovery of PrP^Sc^, including PrP^Sc^ species with low resistance to PK. The 34% prevalence of positive cases harboring detectable PrP^Sc^ was nearly half that of PrP^Sc^-positive Tg mice detected at histopathologic examination (Table 1). Tg mouse resPrP^Sc^ immunoreacted much more readily with the monoclonal antibody 1E4 than with 3F4, as previously reported for VPSPr PrP^Sc^ (*14*). Probed with 1E4, Tg mouse resPrP^Sc^ exhibited a 5-band electrophoretic profile, similar to that of VPSPr (Figure 3). Deglycosylation by PNGase resulted in 3 resPrP^Sc^ bands that co-migrated with the analogous VPSPr bands at ≈20 kDa, ≈17 kDa, and ≈7 kDa (Figure 3) (*15*). No definitely positive WB conducted with standard methods was observed in VPSPr-inoculated Tg(HuPrP129V)×3 and Tg(HuPrP129M)×2 mice (Table 1).

All negative controls, including noninoculated Tg mice and Tg mice inoculated with noninfectious BH, had negative WB. In contrast, WB were positive, with the classical 3-band profile, in all mice from the 3 Tg(HuPrP) lines challenged with BH from different sCJD subtypes used as positive controls (Table 3). WB examination of brains from 3 Tg(HuPrP129V)×8 mice challenged with VPSPr-129VV BH showed no evidence of PrP^Sc^ 35 dpi, indicating that the inoculum was too diluted to be detected or no longer present.

### Second Passage

The second passage yielded a surprising finding. None of the 13 mice belonging to the Tg(HuPrP129V)×8 and Tg(HuPrP129M)×2 lines showed prion-related histologic lesions or had a definitely positive WB upon secondary transmission, even up to nearly 800 dpi (Table 4).

**Table 4:** VPSPr inoculations to Tg(HuPrP): second passage*

Inoculum	Tg(HuPrP)	No. with clinical signs/total	Histology, immunohistochemistry			PrP^Sc^ Western blot	
			No. positive/total	Dpi positive/total†		No. positive/total	Dpi positive/total
Tg(HuPrP129V) inoculated with VPSPr-129VV 2nd‡	(129V)×8§	0/10	0/10	0/616 ± 135		0/10	0/616 ± 135
Tg(HuPrP129M) inoculated with VPSPr-129MM 1st	(129M)×2	0/3	0/3	0/694 ± 51		NA	NA

Dpi, days postinoculation; M, methionine; V, valine; NA, not available; VPSPr, variably protease-sensitive prionopathy associated with homozygosity V or M at codon 129 of the PrP gene (VPSPr-129VV or VPSPr-129MM); †Dpi of positive mice compared with dpi of total mice (including the positive mice). Each value represents the mean ± SD. ‡Identifies the Transgenic mice, inoculated with VPSPr in the first passage, providing the inoculum. §Refers to the presence of V or M at PrP residue 129 and expression level indicated as × normal.

## Discussion

The experiments reported here, which probed transmissibility of VPSPr to Tg mice expressing human PrP^C^, yielded puzzling results. On first passage, all hosts remained asymptomatic, but 54% showed focal deposition of PrP^Sc^ in the form of prion plaques by immunohistochemical analysis. In 34% of animals, small amounts of resPrP^Sc^ were demonstrated by WB (Figures 1, 3). The PrP^Sc^ recovered from affected mice recapitulated the electrophoretic profile and immunoreactivity features of the VPSPr-129VV PrP^Sc^, even after removal of the sugar moiety. However, mouse PrP^Sc^ was apparently more PK-resistant than the PrP^Sc^ from VPSPr-129VV case-patients (Figure 3) (*15*). In contrast, on second passage, all Tg mice were negative at clinical and histopathologic examinations and harbored no PrP^Sc^ that could be definitely identified by WB, even up to 800 dpi.

These findings pose several challenging questions. The first question concerns whether the PrP^Sc^ recovered on first passage represents the residual inoculum rather than de novo PrP^Sc^ generated by conversion of the host’s PrP^C^. We believe this possibility is made unlikely by our experiment showing that the PrP^Sc^ in the VPSPr inoculum was no longer detectable by immunohistochemical and WB analyses in Tg(HuPrP-129VV) mice 35 dpi. Furthermore, histopathologic changes and PrP^Sc^ detection, indicating VPSPr transmission, were observed only when the host and the inoculum were syngenic at PrP codon 129. This genotypic selectivity is not compatible with the notion that the PrP^Sc^ detected in the hosts was from the residual inoculum. Finally, several other studies have shown that PrP^Sc^ associated with the inoculum is rapidly cleared (*12*,*26*–*30*). These observations support the conclusion that histopathologic changes and the PrP^Sc^ recovered in the positive VPSPr-challenged mice result from de novo mouse PrP^Sc^ generated by templated conversion, although at a very low level. The lack of clinical signs in the affected mice can be easily explained by the characteristics and localization of the plaques and of the spongiform degeneration, both of which affected limited regions depopulated of neuronal cells and processes.

Prion plaques similar in type and topography to those we observed have been reported alone or associated with spongiform degeneration in mice challenged with prions of various origins (*9*,*11*,*29*–*32*). In 3 studies, plaque deposits seem to be especially similar to those observed by us. In the first, the plaques were detected on the second passage of vCJD in Tg mice expressing human PrP^C^-129V (Tg152) (*11*,*29*). The second and third studies used Tg mice expressing mouse PrP harboring the P101L variation (101LL mice), which is the equivalent of the P102L human mutation linked to a GSS subtype (*9*,*30*). In the last 2 experiments, Tg101LL mice were challenged with “atypical” P102L GSS (i.e., GSS associated only with 7-kDa PrP^Sc^ PK-resistant fragment rather than with the “typical” P102L GSS that is also associated with the classical 3-band resPrP^Sc^) or with BH from TgGSS-22, a Tg mouse model of GSS in which prion disease spontaneously develops (*9*,*30*). All affected mice of these 3 studies remained asymptomatic like those of our study, further supporting the notion that focal prion plaque deposition in periventricular regions is not sufficient to produce major clinical signs. Despite the common histopathologic features, these mice were associated with electrophoretically distinct PrP^Sc^ species. In the experiment of vCJD transmission, PrP^Sc^ harvested from challenged Tg152 mice showed the classical 3 PK-resistant bands as in vCJD, but they displayed a higher molecular mass than in vCJD (*11*). Minimal quantity of resPrP^Sc^ recovered at ≈30 kDa was detected only in 1 of 5 plaque-harboring 101LL mice challenged with atypical P102L GSS (*9*), whereas 101LL mice inoculated with TgGSS-22 BH showed no detectable PrP^Sc^ by WB (*30*). Although to some extent the variability of the PrP^Sc^ electrophoretic profiles between the experiments with Tg101LL and our experiments might be due to variations in WB methods, the PrP^Sc^ diversity in these 3 studies and in our study suggests that focal plaque formation at the border between the hippocampus and corpus callosum is not strictly PrP^Sc^ strain specific. However, this brief review indicates that peri-hippocampal plaque deposition is preferentially detected in hosts challenged with PrP^Sc^ species that form plaques in the natural disease, such as vCJD, GSS, and VPSPr.

The lack of histologic lesions and PrP^Sc^ on second passage in both Tg(HuPrP129V) and Tg(HuPrP129M) mice challenged with BH from the most severely affected first-passage mice is unusual. However, comparable findings have been reported in at least 2 previous studies. In the first, 101LL mice challenged with affected TgGSS-22 BH (a Tg mouse in which a GSS-like disease spontaneously develops) harbored fewer plaques on second passage than on first, suggesting decreased replication of the seed on second passage (*9*,*30*). In the second study, first passage of BH from cattle affected by bovine spongiform encephalopathy to Tg152 mice (expressing human PrP^C^-129V) resulted in clinical disease associated with diffuse brain deposition of PrP as demonstrated by immunostaining (*11*), even though no resPrP^Sc^ could be demonstrated by WB. On second passage in the same Tg152 mice, no evidence of prion disease could be demonstrated either by PrP immunostaining or WB, as in our study (*11*). However, BH from these negative Tg152 produced a full prion disease after inoculation into wild-type FVB mice. This remarkable finding indicates that prion transmissibility (or infectivity) can be sustained in hosts with no demonstrable prion diseases (according to commonly used methods), and that it can be rescued through passage to an appropriate host (*11*).

Interpreting our findings also in the light of the above data, we propose that normal human PrP^C^ is not a suitable substrate (or mouse brain is not a favorable environment) to sustain conversion to VPSPr PrP^Sc^. Thus, long incubations are required to induce modest and asymptomatic PrP^Sc^ deposition. Nevertheless, the PrP^Sc^ generated in the host on first passage seems to match the conformation of the PrP^Sc^ from the inoculum, based on the finding that PK-resistant PrP^Sc^ fragments of similar size are recovered from host and donor preparations. This also indicates that little or no PrP^Sc^ adaptation has occurred during the first passage (*32*). The apparent failure of the second passage to transmit detectable disease might be due to the inadequate amplification of VPSPr PrP^Sc^ during primary transmission, which would result in a second passage PrP^Sc^ inoculum more diluted than the VPSPr brain extract used in the primary transmission. This line of reasoning raises the possibility that if VPSPr PrP^Sc^ were exposed to a favorable PrP substrate (or brain environment), it might replicate efficiently. This conjecture is reinforced by 2 recent findings. First, preliminary findings show that VPSPr can be transmitted to bank voles more easily than to the Tg(HuPrP) used in the present experiments (*33*). Second, transmissibility of GSS linked to the A117V mutation has been recently demonstrated (*10*). GSS-A117V is commonly viewed as a “classic” GSS subtype, given the distinct features of its phenotype that is characterized by prominent prion plaques with limited spongiform degeneration and a PrP^Sc^ WB profile dominated by a highly PK-resistant fragment of 7–8 kDa (*34*). Early failures to transmit GSS-A117V and the evidence that the presence of the A117V mutation altered the topology of PrP led to the conclusions that 1) GSS-A117V was not transmissible and 2) its underlying pathogenetic mechanism was not based on a PrP^C^-to-PrP^Sc^ conversion process (*8*).

After inoculation with BH from GSS-A117V–affected patients, Tg mice expressing human PrP, harboring the A117V mutation, developed a prion disease associated with a histologic phenotype and PrP^Sc^ that roughly recapitulated those of the human disease. However, only one fourth of the inoculated mice were symptomatic with incubation periods >600 days, whereas more than half harbored plaques, PrP^Sc^, or both (*10*). No second passage was reported. This study shows that an allegedly nontransmissible prion disease can be transmitted (at least at first passage) if a suitable host is selected.

In conclusion, we propose that VPSPr is transmissible and, therefore, is an authentic prion disease. However, transmissibility cannot be sustained through serial passages presumably because human PrP^C^ (or the mouse brain environment) cannot efficiently convert and propagate the VPSPr PrP^Sc^ species. If this is the case, uncovering the properties of human PrP that are required to replicate more efficiently the prion strains associated with VPSPr may help clarify the PrP^Sc^ mode of formation in this intriguing disease.
